# Prognostic Value of CD133 and SOX2 in Advanced Cancer

**DOI:** 10.1155/2019/3905817

**Published:** 2019-01-01

**Authors:** Susu Han, Tao Huang, Xing Wu, Xiyu Wang, Shanshan Liu, Wei Yang, Qi Shi, Hongjia Li, Fenggang Hou

**Affiliations:** ^1^Shanghai Municipal Hospital of Traditional Chinese Medicine, Shanghai University of Traditional Chinese Medicine, 274 Zhijiang Road, Shanghai 200071, China; ^2^The Affiliated Hospital of Ningbo University, Ningbo, Zhejiang 315020, China

## Abstract

**Background:**

The prognostic value of CD133 and SOX2 expression in advanced cancer remains unclear. This study was first conducted to investigate the association between CD133 or SOX2 positivity and clinical outcomes for advanced cancer patients.

**Methods:**

Hazard ratios (HRs) with 95% confidence intervals (95% CIs) were calculated to evaluate the correlation between CD133 or SOX2 positivity and overall survival (OS), disease-free survival (DFS), progression-free survival (PFS), cancer-specific survival (CSS), or recurrence-free survival (RFS) from multivariable analysis. Trial sequential analysis (TSA) was also performed.

**Results:**

13 studies with 1358 cases (CD133) and five studies with 433 cases (SOX2) were identified. CD133 positivity was correlated with worse CSS and OS, but there was no correlation between CD133 positivity and DFS. SOX2 positivity was associated with poor DFS and RFS but was not linked to PFS. Stratified analysis by study source showed that only CD133 positivity can decrease OS for Chinese patients. Stratified analysis by treatment regimens indicated that CD133 positivity was linked to poor OS in patients treated with adjuvant therapy. TSA showed that additional studies were necessary.

**Conclusions:**

CD133 and SOX2 might be associated with worse prognosis in advanced cancer. More prospective studies are strongly needed.

**Impact:**

CD133 and SOX2 may be promising targeted molecular therapy for advanced cancer patients.

## 1. Introduction

Cancer is still one of the most threatening diseases worldwide [1]. Although surgery, chemotherapy, and/or radiotherapy have greatly improved the clinical survival for early cancer patients, therapies for patients with advanced or metastatic cancer still have a major challenge [2]. Improvements in the treatment of advanced or metastatic cancer patients (surgical technique, chemotherapy, radiotherapy, targeted molecular therapy, and immunotherapy regimens) have extended patients' median survival, but such as 5-year overall survival is still poor [3–5]. Thus the development of new and novel therapeutic regimens for advanced or metastatic cancer patients is important.

Increasing evidence has been suggested regarding cancer stem cells (CSCs) in various cancers. The major characteristics of CSCs are the capability of self-renewal, unlimited proliferation and differentiation, and resistance to conventional treatments like chemotherapy or radiation [6, 7]. Recently, some stem cell markers have been described, such as CD44, CD166, EpCAM, CD133, and SOX2 [8–10]. CD133, also named as prominin-1, is a member of pentaspan transmembrane cell surface glycoproteins [11, 12]. Sex-determining region Y-box protein 2 (SOX2), a High Mobility Group (HMG) domain transcription factor, is involved in the regulation of stem cells self-renewal and pluripotency [13]. CD133 expression has been reported and contributes to malignant transformation and chemo- and radioresistance [14]. SOX2 has been studied in some types of human cancers and facilitates tumor initiation and progression [15–17]. Some meta-analyses investigated the prognostic value of CD133 and SOX2 expression in some human cancers [18–21], but the prognostic significance of CD133 and SOX2 expression in advanced cancer patients remains unclear and unknown.

To our knowledge, the expression of CD133 and SOX2 is hitherto undescribed in advanced cancer by a meta-analysis. To clarify the correlation between the expression of stem cell markers (CD133 and SOX2) and the prognosis in advanced or metastatic cancer patients, we investigated the relationship between the expression of these two markers and survival of the samples.

## 2. Materials and Methods

### 2.1. Literature Selection

The present meta-analysis was reported in accordance with the Preferred Reporting Items for Systematic reviews and Meta-Analyses (PRISMA) guideline [22]. The potential studies were identified through searching online databases including PubMed, EMBASE, EBSCO, Web of Science, and Cochrane Library before April 2018 without language restrictions. The main key words and search items were “CD133 OR PROM1 OR prominin-1 OR AC133 antigen OR SOX2 OR Sex determine region Y-box 2 OR SRY box-2 OR SRY-Related HMG-Box Gene 2”, “metastatic OR advanced OR metastasized OR recurrent”, “cancer OR tumor OR carcinoma OR neoplasm”, and “survival OR outcome OR prognosis”. Additional potential articles were also manually searched by the reference lists of the eligible studies.

### 2.2. Eligibility Criteria

Papers identified for the inclusion criteria in this study for the current analysis were as follows: (1) studies reported the patients with advanced, metastatic, or recurrent cancer; (2) studies investigated the prognostic value of expression of CD133 or SOX2; (3) studies presented sufficient data on hazard ratio (HR) with 95% confidence interval (CI) from multivariable analysis for overall survival (OS), disease-free survival (DFS), progression-free survival (PFS), cancer-specific survival (CSS), relapse/recurrence-free survival (RFS), or metastasis-free survival (MFS); (4) unclear data (HR with 95% CI) such as only* P* value with HR or 95% CI, survival data calculated based on the described method [23, 24], or contacting the corresponding author via email to request the available information. If two or more papers used the overlapping or same cancer samples, only the study with the largest patient numbers or the most recent article was selected. Case report, reviews, animal studies, unrelated articles, or survival data using univariable analysis were excluded.

### 2.3. Data Extraction and Study Assessment

The methodology of each eligible study was conducted following REMARK guidelines (Reporting Recommendations for Tumor Marker Prognostic Studies) [25]. 20 criteria were listed in REMARK; each item had scores 0, 1, and 2, with a maximal score of 40 (Table S1). The value was 2 scores when each item was clearly described in the article, 1 score when each item was incompletely defined, and 0 score when each item was not defined or not applicable. We did not define a threshold for the REMARK score of study quality because multivariable survival measures are more valuable than studies using univariable analysis, done in the present meta-analysis. REMARK scores can be used and evaluated for sensitivity analyses. The following information was extracted from eligible studies: first author's name, publication year, study population, study source, mean or median age, type of cancer, detection method, therapy regime, study design, sample type, cut-off value, median or mean follow-up period, survival rate, adjusted variables, and clinical outcomes, etc. All authors resolved the discrepancy when information was controversial.

### 2.4. Data Analysis

To estimate the effect of CD133 or SOX2 expression status on advanced cancer survival (OS, DFS, PFS, CSS, RFS, or MFS of multivariable analysis), the result with an HR >1 demonstrated an unfavorable prognosis, whereas an HR <1 stood for a good prognosis. The Cochran's Q statistic was used to evaluate heterogeneity among the included studies [26]. The random-effects model (DerSimonian-Laird) was used in the meta-analysis (heterogeneity:* P *< 0.1) [27, 28]. For the results (> seven studies) with substantial heterogeneity, subgroup analyses based on tumor type, study source, survival rate, sample type, age (years), testing method, and study center design were performed to explain the potential heterogeneity and different strength of the association between subgroups. If all relevant* P* values of heterogeneity were greater than 0.1 among different subgroups, it indicates the source of heterogeneity from a subgroup variable. The Egger's and Begg's funnel plots were used to evaluate publication bias [29, 30]. Pooled data were analyzed using Stata software, version 12.0 (Stata Corp., College Station, TX, USA).

### 2.5. Trial Sequential Analysis

In the meta-analysis involving a small number of participants, random errors can lead to spurious results [31, 32]. Trial sequential analysis (TSA) was conducted to control random errors and to estimate the required study population [33]. The optimal a priori anticipated information size (APIS) method was set in our study. We calculated diversity-adjusted TSA based on the relative risk reduction (RRR) of 20%, the prespecified type I error of 5%, and the type II error (20% or 10%). We also calculated diversity-adjusted TSA based on a RRR of 15%, the prespecified type I error (*α*) of 5%, and a type II error (*β*) of 20%. Monitoring boundaries are applied to decide whether a clinical trial could be terminated early. When the cumulative Z curve was more than the trial sequential monitoring boundary or required information size (RIS) boundary, it suggested the firm evidence. Otherwise, more clinical studies are needed. Meta-analysis of HR estimates was performed using Stata software, version 12.0 (Stata Corp., College Station, TX, USA) and R software, version 3.4.2 (The R Foundation for Statistical Computing, Vienna, Austria).

## 3. Results

### 3.1. Study Characteristics

**Figure 1:**
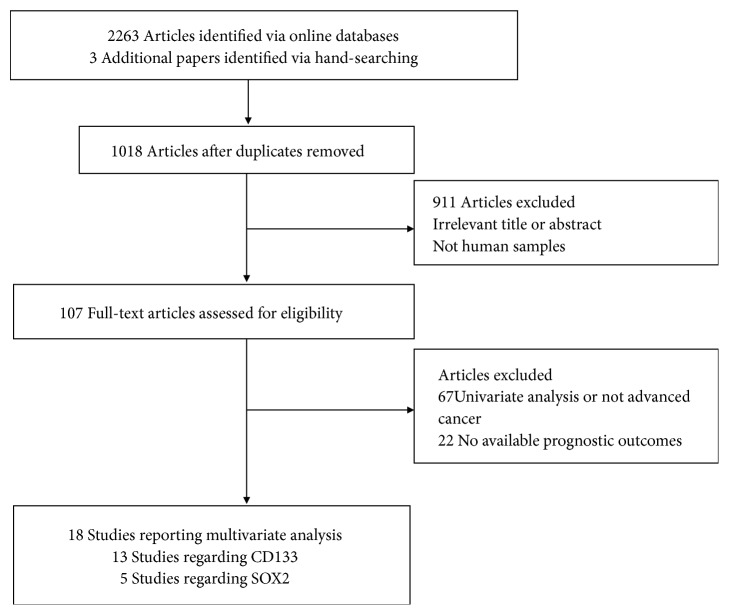
Flow chart for identification of eligible studies.

Flowchart describing the study selection process is shown in Figure 1. After the described inclusion criteria, 18 eligible studies involving 1791 advanced cancer patients were selected for the current meta-analysis [34–51]. Of these studies, 13 studies published from 2006 to 2017 (one prospective study and 12 retrospective studies) evaluated the prognostic role of CD133 positivity [35, 37, 38, 40, 42, 44–51], including 1358 cases. Five studies (one prospective study and four retrospective studies) assessed the prognostic role of SOX2 positivity [34, 36, 39, 41, 43], including 433 cases. The mean REMARK scores were 21, with a range from 12 to 28. Most studies (78%) reported patients treated with adjuvant therapy. All articles published were from 2006 to 2017, and six studies were conducted in China, six studies in Japan, one study in Korea, and the remaining five studies in Europe. The characteristics of the eligible studies using multivariable analysis are listed in Table 1 and Table S2.

### 3.2. Association between CD133 Positive Expression and the Prognosis

**Figure 2:**
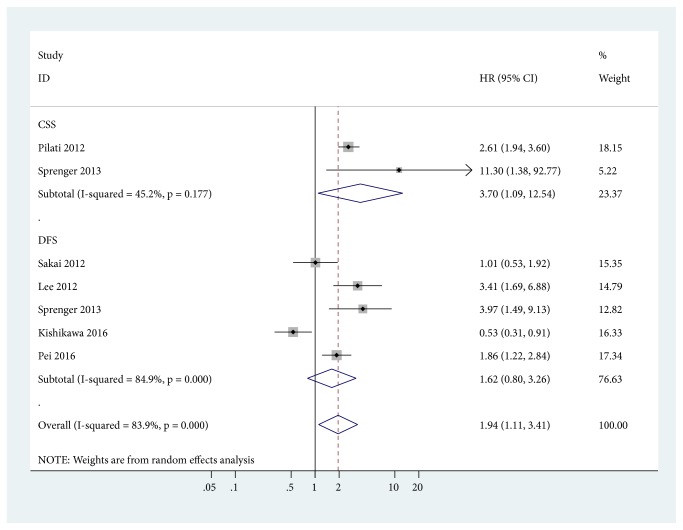
Forest plot for the correlation between CD133 positive expression and cancer-specific survival (CSS) and disease-free survival (DFS).

The pooled data from two studies involving 176 advanced cancer patients showed that CD133 positive expression was associated with a worse cancer-specific survival (CSS) (HR = 3.70, 95% CI = 1.09-12.54,* P* = 0.036) (Figure 2). Data from five studies involving 729 patients with advanced cancer demonstrated no association between CD133 positive expression and DFS (HR = 1.62, 95% CI = 0.80-3.26,* P* = 0.178) (Figure 2).

**Figure 3:**
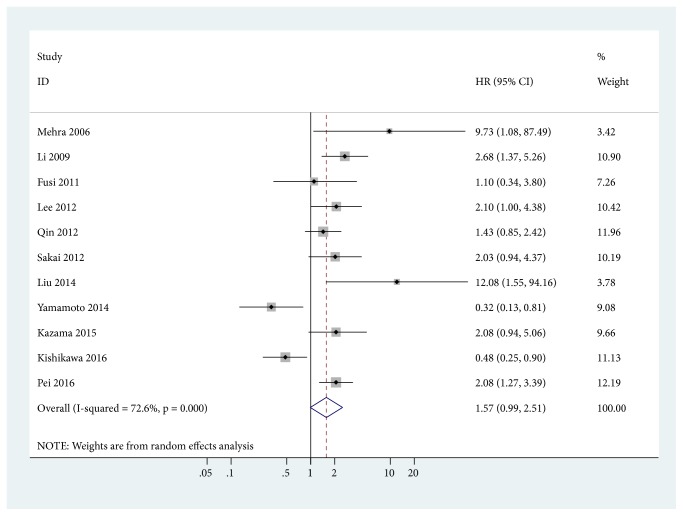
Forest plot for the correlation between CD133 positive expression and overall survival (OS).

11 studies with 1182 cases were included in the final analysis of CD133 positivity and OS. Data showed that CD133 positivity was slightly correlated with an unfavorable OS (HR = 1.57, 95% CI = 0.99-2.51,* P* = 0.057) (Figure 3).

### 3.3. Subgroup and Sensitivity Analyses of CD133 Positive Expression in OS

We summarized the results of the subgroup analyses among several related clinical parameters (tumor type, study source, survival rate, sample type, age (years), testing method, study center design, treatment regimens, and sample size) for OS in Table 2. All* P* values of heterogeneity were not more than 0.1 between different subgroups; subgroup analyses did not find the potential sources of heterogeneity.

Based on tumor type, significant difference was not found in 848 patients with colorectal cancer (six studies: HR = 1.27, 95% CI = 0.64-2.50,* P* = 0.493), 152 patients with ovarian cancer (two studies: HR = 3.27, 95% CI = 0.43-25.03,* P* = 0.254), and 32 patients with melanoma (one study: HR = 1.1, 95% CI = 0.34-3.8). There was statistical significance in patients with 50 cancer patients with bone metastases (one study: HR = 9.73, 95% CI = 1.08-87.49) and 100 patients with gastric cancer (one study: HR = 2.097, 95% CI = 1.003-4.383).

Subgroup analysis by treatment regimens indicated that CD133 positivity was slightly linked to poor OS in patients treated with adjuvant therapy (4 studies with 309 cases: HR = 1.91, 95% CI = 1.08-3.39,* P* = 0.026). Subgroup analysis of study source showed that only Chinese with CD133 positivity was significantly correlated with a worse OS (four studies with 579 cases: HR = 2.12, 95% CI = 1.35-3.33,* P *= 0.001). Subgroup analysis of survival rate indicated that CD133 positivity was significantly related to a less than 3-year OS (two studies with 79 cases: HR = 10.92, 95% CI = 2.44-48.96,* P *= 0.002). Stratified analysis by age demonstrated that CD133 positivity was significantly associated with shorter OS in patients aged more than 60 years (two studies with 238 cases: HR = 2.09, 95% CI = 1.20-3.64,* P *= 0.009). Significant difference was not noted between other subgroup analyses (sample type, study center design, and sample size) and CD133 positivity (Table 2).

Sensitivity analysis was performed by omitting an individual study by turn to detect the robustness of the result. The result showed that two studies conducted by Yamamoto 2014 et al. [42] and Kishikawa 2016 et al. [37] in Japan significantly affected the pooled HR value, with the significant HR (2.02, 95% CI = 1.56-2.60,* P* < 0.001) and no evidence of heterogeneity (*P* = 0.413).

### 3.4. Association between SOX2 Positive Expression and the Prognosis

**Figure 4:**
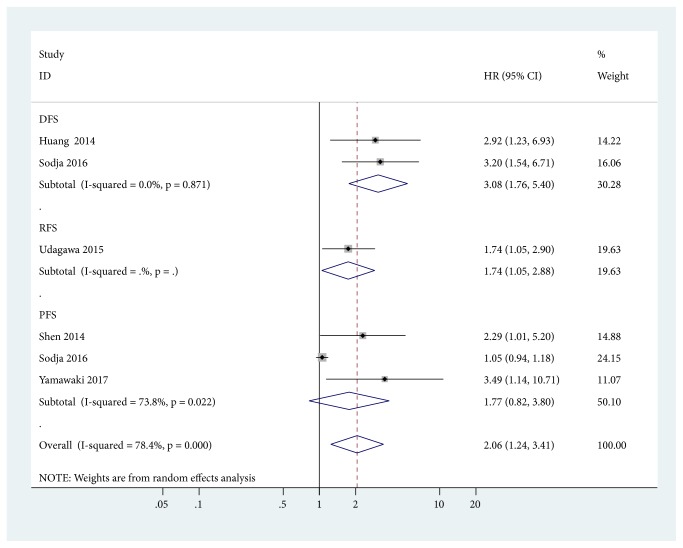
Forest plot for the association between SOX2 positivity and disease-free survival (DFS), relapse/recurrence-free survival (RFS), and progression-free survival (PFS).

SOX2 positivity was associated with worse DFS (two studies with 157 cases: HR = 3.08, 95% CI = 1.76-5.40,* P *< 0.001) and RFS (one study with 113 cases: HR = 1.736, 95% CI = 1.055-2.901,* P *= 0.033), but no relationship was found between SOX2 positivity and PFS (three studies with 213 cases: HR = 1.77, 95% CI = 0.82-3.80,* P *= 0.145) (Figure 4).

### 3.5. Publication Bias

Publication bias was detected for OS and DFS of CD133 positive expression. No evidence of publication bias was noted using Egger's test (*P* = 0.564 > 0.05) and Begg's test (*P* = 0.876 > 0.05) in OS (Figure S1). Moreover, we did not find publication bias for DFS of CD133 positive expression (*P* > 0.1) (Figure S1).

### 3.6. TSA

**Figure 5:**
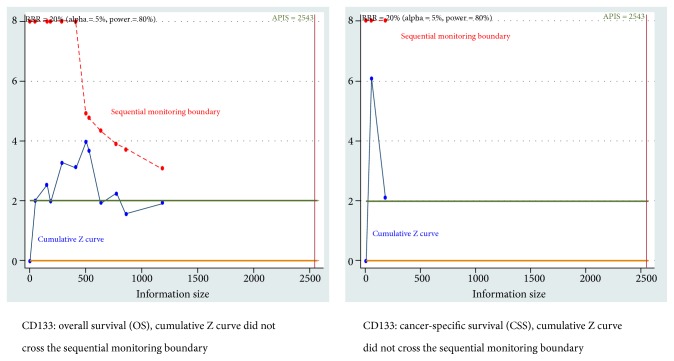
Trial sequential analysis (TSA) for cancer-specific survival (CSS) and overall survival (OS) of CD133 positive expression (*α* = 5%, *β* = 20%, and the relative risk reduction (RRR) = 20%).

When the prespecified type I error *α* (5%), a RRR of 20%, and a type II error *β* of 20% (80% power) were set, the TSA showed that cumulative Z curve did not cross the sequential monitoring boundary for CSS and OS of CD133 positive expression (Figure 5). For DFS of SOX2 positivity, cumulative Z curve was not more than the sequential monitoring boundary (Figure S2A). For positive results of OS of CD133 positivity among subgroups, the TSA also demonstrated that cumulative Z curve did not cross the trial sequential monitoring boundary (Table 2).

**Figure 6:**
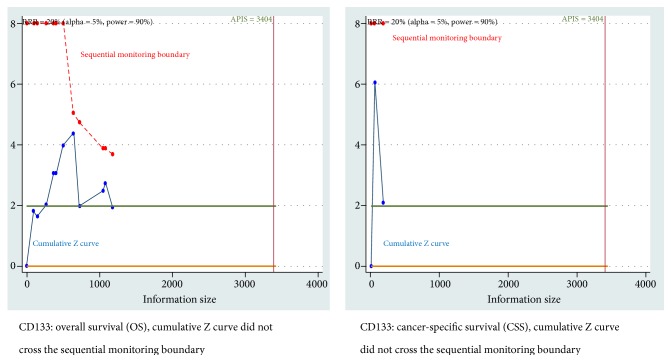
Trial sequential analysis (TSA) for cancer-specific survival (CSS) and overall survival (OS) of CD133 positive expression (*α* = 5%, *β* = 10%, and the relative risk reduction (RRR) = 20%).

When the type I error of 5%, a RRR of 20%, and a type II error of 10% (90% power) were used, TSA also demonstrated that the cumulative Z curve did not reach the sequential monitoring boundary between CD133 positivity and CSS and OS (Figure 6). The TSA showed that the cumulative Z curve did not cross the trial sequential monitoring boundary between SOX2 positivity and DFS (Figure S2B).

**Figure 7:**
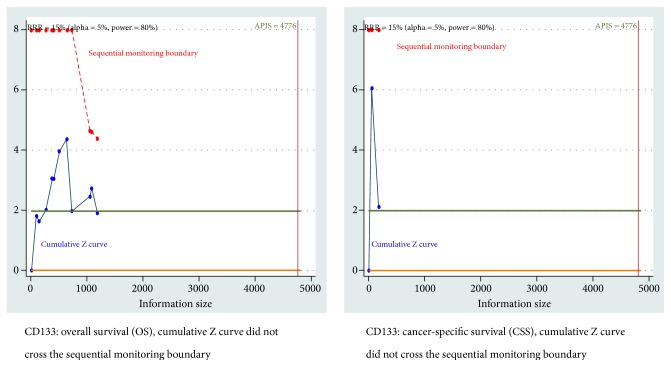
Trial sequential analysis (TSA) for cancer-specific survival (CSS) and overall survival (OS) of CD133 positive expression (*α* = 5%, *β* = 20%, and the relative risk reduction (RRR) = 15%).

When the type I error of 5%, type II error of 20%, and a more conservative RRR of 15% were set, the results remained consistent, and the TSA also showed that cumulative Z curve did not reach the trial sequential monitoring boundary (Figure 7 and Figure S2C).

## 4. Discussion

CSCs, a small subpopulation of tumor cells, drive the growth and progression of cancers [52]. More importantly, CSCs are considered to be involved in chemotherapy/radiotherapy resistance, metastasis, and postoperative recurrence [53, 54]. Some meta-analyses showed that CD133 was a biomarker of putative CSCs in many solid tumors and its positivity may be associated with poor overall survival in nonsmall-cell lung cancer [55], worse prognosis in patients with glioblastoma [20], and reduced overall survival in colorectal cancer [56]. SOX2 expression may be correlated with better overall survival in nonsmall cell lung cancer [21], but worse overall survival in head and neck cancer [57]. However, some results were contradictory, for example, patients with CD133-positive is correlated with a better prognosis in colorectal liver metastasis [42]. Patients with CD133-positive are associated with an unfavorable prognosis in advanced colorectal cancer [35]. The conventional prognostic factors such as tumor stage or grade could not well predict clinical outcome based on an individual basis [58]. To date, there are still no effective markers available for the prognosis of patients with advanced cancer. Therefore, it remains important to better understand the characteristics of CSCs, CD133, and SOX2 for valuable therapeutic and prognostic targets in clinical practice to predict disease outcomes in advanced or metastatic cancer patients. In our meta-analysis, we have attempted to estimate the prognostic effect of CSCs, CD133, and SOX2 using multivariable analysis in patients with advanced or metastatic cancer.

Chemotherapy and radiotherapy are major treatment strategies to eliminate cancer cells, but chemoresistance, radioresistance, and cancer recurrence are major obstacles for the long-term survival of cancer patients [59, 60]. Recent studies show that CSCs are resistant to chemotherapy and radiotherapy and targeting CSCs may become a promising opportunity to cure patients with cancer [54, 61]. The studies of 78% (14 studies) reported patients with adjuvant therapy such as chemotherapy and radiotherapy in this meta-analysis. According to a comprehensive analysis of published studies (CD133: 13 studies with 1358 patients and SOX2: five studies with 433 patients). We found that patients with CD133-positive advanced cancer was correlated with poorer CSS (HR = 3.70,* P* = 0.036) and showed a trend towards poor OS (HR = 1.57,* P* = 0.057), but no relationship was reported between CD133 positivity and DFS (HR = 1.62,* P* = 0.178). For the analyses of CD133 in OS, we performed sensitivity and subgroup analyses. The removal of the study by Yamamoto 2014 [42] used blinding of the detection and the removal of the study by Kishikawa 2016 [37] did not report blinding of the detection (Table 1). We did not find that the possible factors and reasons can influence the pooled HR of OS in CD133. Because these two retrospective studies [37, 42] reported that CD133 positivity was linked to favorable OS. SOX2 positivity was related to shorter DFS (HR = 3.08,* P *< 0.001) and RFS (HR = 1.736,* P *= 0.033), but SOX2 positivity was not correlated with PFS (HR = 1.77,* P *= 0.145). In addition, no publication bias was observed in OS and DFS of CD133. These positive results were further proven by TSA, and the data suggested that additional clinical trials were needed to confirm these conclusions.

We further performed subgroup analyses of CD133 expression stratified by cancer type, study source, survival rate, sample type, age (years), testing method, study center design, and sample size in OS. Subgroup analysis by cancer type showed that CD133 expression was associated with shorter OS in cancer with bone metastases and gastric cancer but no relationship in colorectal cancer, ovarian cancer, and melanoma. Stratified analysis by study source indicated that only CD133 positivity could significantly reduce OS in Chinese patients (HR = 2.12,* P *= 0.001), suggesting that CD133 may play a more important role in the prognosis of advanced cancer for Chinese. Stratified analysis by survival rate showed that only CD133 positivity might significantly decrease OS in patients with < 3-year survival rate (HR = 10.92,* P *= 0.002), which suggested that the expression of CD133 may be correlated with shorter OS within 3 years. Subgroup analysis by age indicated that only CD133 positivity can significantly shorten OS in patients aged more than 60 years (HR = 2.09,* P *= 0.009), suggesting that CD133 may play a more key role in the prognosis for elderly patients. However, no significant difference was found between CD133 positivity and other subgroups such as sample type, study center design, and sample size. We further used TSA to achieve more meaningful results among different subgroups. TSA suggested that the available sample data were insufficient to draw firm conclusions regarding the expression of CD133 to OS.

Our meta-analysis had some limitations. First, the number of the included studies was not very large and some of these eligible studies had small sample sizes. TSA confirmed that cumulative Z curve did not cross the sequential monitoring boundary. Thus, more trials are needed for more reliable results. Second, studies were mainly conducted in China, Japan, and Europe; thus, other study sources (USA) are lacking. Third, most studies were of retrospective design; only two studies were of prospective design. Additional prospective clinical studies (such as blinded detection of CD133 and SOX2 expression) are essential to obtain more firm results in different cancer types, such as colorectal, lung, breast, and head-neck cancer. Finally, there was considerable heterogeneity in this meta-analysis. Although we analyzed several factors that may influence heterogeneity, these variables could not clearly explain the sources of heterogeneity. Thus, clinical practice should interpret our results with caution.

To conclude, our meta-analysis showed that CD133-positive expression may be associated with worse CSS and OS. Subgroup analysis by tumor type showed that CD133 positivity was linked to worse OS in cancer with bone metastases and gastric cancer. Subgroup analysis by study source demonstrated that only CD133 positivity was related to poor OS for Chinese. Subgroup analysis by survival rate showed that CD133 positivity was correlated with a less than 3-year OS. Subgroup analysis by age demonstrated that the expression of CD133 was associated with shorter OS in patients > 60 years. SOX2 positivity may be related to poor DFS and RFS. Further TSA suggested the need for additional clinical studies. Herein, more high-quality prospective studies are essential to obtain more reliable evidence and help stratify advanced cancer patients who can benefit from different therapies.

## Figures and Tables

**Table 1:** Main characteristics of studies included in the meta-analysis.

First author	Study source	Age	Testing method	Cancer type	Study design	Specimen type	Cases	Survival rate	Outcomes	Therapy
Mehra 2006	The Netherlands	NA	NASBA	Cancer with bone metastases	Retrospective, multicentre	Blood	50	< 3 years	OS	Part (adjuvant therapy)

Li 2009	China	NA	IHC	Advanced colon carcinoma	Retrospective, single-center	Tissue	104	5 years	OS	Adjuvant chemotherapy

Fusi 2011	Germany	54	Flow cytometry analysis	Metastatic melanoma	Retrospective, single-center	Blood	32	NA	OS	Neoadjuvant chemotherapy

Pilati 2012	Italy	63	qRT-PCR	Colorectal liver metastasis	Retrospective, single-center	Blood	50	3 years	CSS	Surgery and chemotherapy

Sakai 2012	Japan	NA	IHC	Colorectal cancer with liver metastasis	Retrospective, single-center	Tissue	92	3 years	OS, DFS	Surgery

Qin 2012	China	NA	IHC	Advanced serous ovarian cancer	Retrospective, multicentre	Tissue	123	NA	OS	Adjuvant chemotherapy

Lee 2012	Korea	61.5	IHC	Advanced gastric cancer	Retrospective, single-center	Tissue	100	5 years	OS, DFS	Surgery and adjuvant chemotherapy

Sprenger 2013	Germany	63	IHC, blind	Advanced rectal adenocarcinoma	Prospective, multicentre	Tissue	126	NA	CSS, DFS	Surgery and radiochemotherapy

Yamamoto 2014	Japan	NA	IHC, blind	Colorectal cancer liver metastasis	Retrospective, single-center	Tissue	103	5 years	OS	Surgery and chemotherapy

Liu 2014	China	57	IHC, blind	Epithelial ovarian cancer with central nervous system metastasis	Retrospective, single-center	Tissue	29	< 3 years	OS	Surgery and adjuvant therapy

Kazama 2015	Japan	67.1	IHC	Colorectal cancer with lymph node metastasis	Retrospective, single-center	Tissue	138	> 5 years	OS	Surgery and adjuvant chemotherapy

Kishikawa 2016	Japan	59.4	IHC	Colorectal cancer with synchronous liver metastases	Retrospective, single-center	Tissue	88	NA	OS, DFS	Surgery and adjuvant chemotherapy

Pei 2016	China	NA	IHC, blind	Advanced colorectal cancer	Retrospective, single-center	Tissue	323	NA	OS, DFS	Surgery and adjuvant chemotherapy

Huang 2014	China	NA	IHC, blind	Breast cancer with axillary lymph nodes	Retrospective, multicentre	Tissue	107	NA	DFS	NA

Shen 2014	China	51	IHC, blind	Advanced cervical squamous cell carcinoma	Retrospective, multicentre	Tissue	132	5 years	PFS	Radiotherapy

Udagawa 2015	Japan	66	IHC	Lung squamous cell carcinoma with lymph node metastasis	Retrospective, single-center	Tissue	113	NA	RFS	Surgery

Sodja 2016	Slovenia	65	qRT-PCR	Advanced small-cell lung cancer	Prospective, single-center	Blood	50	NA	DFS, PFS	Chemotherapy

Yamawaki 2017	Japan	NA	IHC	Advanced endometrial cancer	Retrospective, single-center	Tissue	31	NA	PFS	NA

NA: not applicable; NASBA: nuclear acid sequence-based amplification; IHC: immunohistochemistry; qRT-PCR: Real-Time Quantitative PCR; OS: overall survival; DFS: disease-free survival; PFS: progression-free survival; CSS: cancer-specific survival; RFS: recurrence-free survival (RFS).

**Table 2:** Subgroup analyses of CD133 positivity in overall survival (OS).

Factors	Subgroups	Studies	HR with 95% CI	Heterogeneity (*P*)	P value	Cases	TSA
Tumor type	Colorectal cancer	6	1.27 (0.64-2.50)	< 0.001	0.493	848	
	Ovarian cancer	2	3.27 (0.43-25.03)	0.049	0.254	152	
	Melanoma	1	1.1 (0.34-3.8)	NA	> 0.05	32	
	Cancer with bone metastases	1	9.73 (1.08-87.49)	NA	< 0.05	50	More
	Gastric cancer	1	2.097 (1.003-4.383)	NA	< 0.05	100	More
							
Study source	Japanese	4	0.90 (0.36-2.26)	0.001	0.823	421	
	Chinese	4	2.12 (1.35-3.33)	0.154	0.001	579	More
	Others	3	2.07 (0.90-4.78)	0.229	0.089	182	
							
Survival rate	5 years	3	1.26 (0.38-4.17)	0.001	0.703	307	
	< 3 years	2	10.92 (2.44-48.96)	0.888	0.002	79	More
	Others	6	1.38 (0.85-2.26)	0.01	0.197	796	
							
Sample type	Tissue	9	1.51 (0.92-2.49)	< 0.001	0.103	1100	
	Blood	2	2.68 (0.33-21.83)	0.088	0.358	82	
							
Age (years)	> 60	2	2.09 (1.20-3.64)	0.989	0.009	238	More
	≤ 60	3	1.40 (0.31-6.27)	0.01	0.661	149	
	NA	6	1.64 (0.92-2.91)	0.003	0.093	795	
							
Treatment regimens	Adjuvant therapy	4	1.91 (1.08-3.39)	0.173	0.026	309	More
	Surgery and adjuvant therapy	6	1.34 (0.62-2.93)	< 0.001	0.457	781	
							
Testing method	Blind	3	1.64 (0.32-8.37)	< 0.001	0.553	455	
	NA	8	1.62 (1.00-2.63)	0.005	0.048	727	More
							
Study center design	Multicentre	2	2.74 (0.46-16.19)	0.097	0.266	173	
	Single-center	8	1.54 (0.85-2.79)	< 0.001	0.154	977	
	NA	1	1.1 (0.34-3.8)	NA	> 0.05	32	
							
Sample size	≥ 100	6	1.58 (0.97-2.57)	0.007	0.066	891	
	< 100	5	1.93 (0.66-5.65)	0.001	0.232	291	

HR: hazard ratio; 95% CI: 95% confidence interval; NA: not applicable; TSA: trial sequential analysis.

## Data Availability

The data used to support the findings of this study are included within the article.

## Supplementary Materials

Supplementary 1 Table S1: REMARK guidelines.

Supplementary 2 Table S2: Detailed characteristics of studies included in the meta-analysis.

Supplementary 3 Figure S1: Publication bias using Egger's and Begg's tests for overall survival (OS) and disease-free survival (DFS) of CD133 positive expression.

Supplementary 4 Figure S2: Trial sequential analysis (TSA) for disease-free survival (DFS) of SOX2 positivity.
