# Supplementary material for: Prognostic Value of CD133 and SOX2 in Advanced Cancer
Source: J Oncol. 2019 Jan 1;2019:3905817. doi: 10.1155/2019/3905817 (PMC6332999; doi:10.1155/2019/3905817)
Supplement: Supplementary 1 — Table S1: REMARK guidelines. [file 3905817.f1.docx]

**Table S1 REMARK guidelines**

| **Author** | **1** | **2** | **3** | **4** | **5** | **6** | **7** | **8** | **9** | **10** | **11** | **12** | **13** | **14** | **15** | **16** | **17** | **18** | **19** | **20** | **Total** |
| --- | --- | --- | --- | --- | --- | --- | --- | --- | --- | --- | --- | --- | --- | --- | --- | --- | --- | --- | --- | --- | --- |
| Mehra 2006 | 2 | 1 | 1 | 2 | 1 | 1 | 0 | 2 | 0 | 1 | 1 | 0 | 1 | 2 | 2 | 2 | 2 | 0 | 2 | 2 | 25 |
| Li 2009 | 2 | 1 | 2 | 1 | 1 | 1 | 2 | 2 | 0 | 1 | 2 | 1 | 1 | 2 | 1 | 1 | 1 | 0 | 1 | 1 | 24 |
| Fusi 2011 | 2 | 0 | 0 | 1 | 1 | 0 | 2 | 2 | 0 | 1 | 2 | 1 | 1 | 2 | 1 | 2 | 1 | 0 | 1 | 1 | 21 |
| Pilati 2012 | 0 | 2 | 1 | 1 | 1 | 1 | 2 | 2 | 2 | 1 | 1 | 1 | 1 | 0 | 2 | 2 | 1 | 1 | 1 | 2 | 25 |
| Sakai 2012 | 0 | 1 | 1 | 1 | 1 | 1 | 0 | 0 | 0 | 1 | 0 | 0 | 1 | 0 | 1 | 2 | 2 | 0 | 0 | 0 | 12 |
| Qin 2012 | 1 | 1 | 1 | 1 | 1 | 1 | 2 | 0 | 0 | 1 | 0 | 0 | 1 | 2 | 2 | 2 | 2 | 0 | 1 | 0 | 19 |
| Lee 2012 | 2 | 1 | 2 | 1 | 1 | 1 | 0 | 0 | 0 | 1 | 0 | 0 | 1 | 2 | 1 | 1 | 1 | 0 | 1 | 1 | 17 |
| Sprenger 2013 | 2 | 2 | 2 | 1 | 1 | 1 | 2 | 0 | 0 | 1 | 1 | 2 | 1 | 2 | 1 | 2 | 2 | 0 | 1 | 1 | 25 |
| Yamamoto 2014 | 2 | 2 | 1 | 1 | 1 | 1 | 0 | 0 | 0 | 1 | 0 | 0 | 1 | 0 | 2 | 2 | 2 | 0 | 1 | 1 | 18 |
| Liu 2014 | 1 | 2 | 1 | 1 | 1 | 1 | 2 | 0 | 0 | 1 | 1 | 1 | 1 | 2 | 2 | 2 | 2 | 0 | 2 | 2 | 25 |
| Kazama 2015 | 2 | 2 | 1 | 1 | 1 | 1 | 2 | 1 | 0 | 1 | 0 | 0 | 1 | 2 | 1 | 2 | 2 | 0 | 2 | 1 | 23 |
| Kishikawa 2016 | 2 | 1 | 1 | 1 | 1 | 1 | 0 | 0 | 0 | 1 | 0 | 0 | 1 | 2 | 1 | 2 | 1 | 0 | 2 | 1 | 18 |
| Pei 2016 | 2 | 2 | 1 | 1 | 1 | 1 | 2 | 1 | 0 | 1 | 0 | 0 | 1 | 2 | 2 | 2 | 2 | 0 | 1 | 0 | 22 |
| Udagawa 2015 | 0 | 2 | 1 | 1 | 1 | 1 | 2 | 0 | 0 | 1 | 0 | 0 | 2 | 0 | 2 | 0 | 2 | 0 | 1 | 0 | 16 |
| Huang 2014 | 2 | 1 | 0 | 1 | 1 | 1 | 0 | 2 | 0 | 1 | 0 | 1 | 0 | 0 | 1 | 2 | 1 | 0 | 2 | 1 | 17 |
| Shen 2014 | 2 | 2 | 2 | 1 | 1 | 1 | 2 | 2 | 0 | 1 | 1 | 0 | 1 | 2 | 2 | 1 | 1 | 0 | 1 | 2 | 25 |
| Sodja 2016 | 2 | 1 | 2 | 1 | 1 | 1 | 2 | 2 | 0 | 1 | 2 | 1 | 1 | 2 | 2 | 2 | 1 | 0 | 2 | 2 | 28 |
| Yamawaki 2017 | 2 | 1 | 0 | 1 | 1 | 1 | 0 | 0 | 0 | 1 | 0 | 0 | 1 | 2 | 2 | 0 | 0 | 0 | 1 | 1 | 14 |
