# Supplementary material for: Prognostic Value of CD133 and SOX2 in Advanced Cancer
Source: J Oncol. 2019 Jan 1;2019:3905817. doi: 10.1155/2019/3905817 (PMC6332999; doi:10.1155/2019/3905817)
Supplement: Supplementary 2 — Table S2: Detailed characteristics of studies included in the meta-analysis. [file 3905817.f2.docx]

**Table S2 Detailed characteristics of studies included in the meta-analysis**

|  | First author | Year | Study source | Median follow-up time | Age | Testing method | Cancer type | Study design | Specimen type | Cases | Therapy | Staining  patterns | Cut off | Survival rate | Adjusted variables | Clinical outcomes | REMARK scores |
| --- | --- | --- | --- | --- | --- | --- | --- | --- | --- | --- | --- | --- | --- | --- | --- | --- | --- |
| CD133 positivity |  |  |  |  |  |  |  |  |  |  |  |  |  |  |  |  |  |
|  | Mehra 2006 | 2006 | The Netherlands | 16 months (3-33) | NA | Nuclear acid sequence-based amplification | Cancer with bone metastases | Retrospective, multicentre | Blood | 50 | Part (adjuvant therapy) | NA | mRNA  200 copies | < 3 years | Hemoglobin, leukocytes, lactate dehydrogenase, alkaline phosphatase, and treatment-naI¨ve | OS | 25 |
|  | Li 2009 | 2009 | China | NA | NA | IHC | Advanced colon carcinoma | Retrospective, single-center | Paraffin-embedded  tumor specimens | 104 | Adjuvant  chemotherapy | Abcam, Cambridge, UK; dilution 1:150 | Cell membrane 5% | 5 years | Gender, age, sites of primary mass, the T stages, pathological classifications, and tumor grades | OS | 24 |
|  | Fusi 2011 | 2011 | Germany | NA | 54 | Flow cytometry analysis | Metastatic melanoma | Retrospective, single-center | Blood | 32 | Neoadjuvant chemotherapy | Clone 293C3, product number 130-090-826, Miltenyi  Biotec, Bergisch Gladbach, Germany | 0% | NA | LDH levels, tumor burden, and number of metastatic sites | OS | 21 |
|  | Pilati 2012 | 2012 | Italy | 36 months (23-46) | 63 | Real-Time Quantitative PCR | Colorectal liver metastasis | Retrospective, single-center | Blood | 50 | Surgery + chemotherapy | ABI PRISM 7500 Sequence Detection system | NA | 3 years | Age, sex, clinical risk score, and preoperative (neoadjuvant) chemotherapy | CSS | 25 |
|  | Sakai 2012 | 2012 | Japan | 38 months (8-115) | NA | IHC | Colorectal cancer with liver metastasis | Retrospective, single-center | Paraffin-embedded tumor specimens | 92 | Surgery | ab19898, Abcam, Cambridge, UK; dilution 1:500 | 5% | 3 years | Timing of metastases, tumor size, primary lymph node involvement, and number of metastases | OS, DFS | 12 |
|  | Qin 2012 | 2012 | China | NA | NA | IHC | Advanced serous ovarian cancer | Retrospective, multicentre | Paraffin-embedded tumor specimens | 123 | Adjuvant chemotherapy | Abcam; dilution 1:100 | Cytoplasm and/or membrane 10% | NA | Age, grade, stage, response to treatment, EGFR, VEGF, and MVD | OS | 19 |
|  | Lee 2012 | 2012 | Korea | Mean 46.9 months (0-115) | 61.5 | IHC | Advanced gastric cancer | Retrospective, single-center | Paraffin-embedded tumor specimens | 100 | Surgery + adjuvant chemotherapy | Abnova., Taipei, Taiwan; dilution 1:200 | Cell membrane/cytoplasm 6 scores | 5 years | Tumor stage, lymphatic,vascular, and neural invasion | OS, DFS | 17 |
|  | Sprenger 2013 | 2013 | Germany | 45 months | 63 | IHC, blind | Advanced rectal adenocarcinoma | Prospective, multicentre | Paraffin-embedded tumor specimens | 126 | Surgery + radiochemotherapy | clone C24B9; Cell Signaling Technology Inc., Danvers, Mass; dilution 1:100 | Cell membrane/cytoplasm 40% | NA | Preoperative therapy, UICC pathologic tumor classification, UICC pathologic lymph node status, and downstaging | CSS, DFS | 25 |
|  | Yamamoto 2014 | 2014 | Japan | NA | NA | IHC, blind | Colorectal cancer liver metastasis | Retrospective, single-center | Paraffin-embedded tumor specimens | 103 | Surgery + chemotherapy | AC133; Miltenyi Biotec, Auburn, CA, USA; dilution 1:40 | Cell membrane/cytoplasm 10% | 5 years | Number, maximum size, doubling time, prehepatectomy chemotherapy, and adjuvant chemotherapy | OS | 18 |
|  | Liu 2014 | 2014 | China | 13.2 months (6.9-19.5) | 57 | IHC, blind | Epithelial ovarian cancer with central nervous system metastasis | Retrospective, single-center | Tissue | 29 | Surgery + adjuvant therapy | Abcam, Cambridge, UK | Cell membrane/cytoplasm 0% | < 3 years | Platinum resistance, multiple CNS metastases, and multimodal therapy | OS | 25 |
|  | Kazama 2015 | 2015 | Japan | 10.7 years | 67.1 | IHC | Colorectal cancer with lymph node metastasis | Retrospective, single-center | Paraffin-embedded tumor specimens | 138 | Surgery + adjuvant chemotherapy | AC133; Miltenyi Biotec, Auburn, CA, USA; dilution 1:100 | Cell surface 5% | > 5 years | Lymphatic involvement, venous involvement, number of lymph node metastasis, and aduvant therapy | OS | 23 |
|  | Kishikawa 2016 | 2016 | Japan | 52.5 months | 59.4 | IHC | Colorectal cancer with synchronous liver metastases | Retrospective, single-center | Paraffin-embedded tumor specimens | 88 | Surgery + adjuvant chemotherapy | AC133; Miltenyi Biotec, Auburn, CA, USA; dilution 1:100 | Cell surface 5% | NA | Depth of invasion, regional lymph node metastasis, histological classification, lymphatic invasion, venous invasion, number of liver metastasis, diameter of liver metastasis | OS, DFS | 18 |
|  | Pei 2016 | 2016 | China | NA | NA | IHC, blind | Advanced colorectal cancer | Retrospective, single-center | Paraffin-embedded tumor specimens | 323 | Surgery + adjuvant chemotherapy | 18470-1-AP, Proteintech, China; dilution 1:200 | Membrane 4 scores | NA | IVE, gross tumor morphology, histologic type, pN, sub-class of stage III, and serum CEA, CA199, and CA242 levels | OS, DFS | 22 |
| SOX2 positivity |  |  |  |  |  |  |  |  |  |  |  |  |  |  |  |  |  |
|  | Huang 2014 | 2014 | China | Mean 38 months (1-146) | NA | IHC, blind | Breast cancer with axillary lymph nodes | Retrospective, multicentre | Paraffin-embedded tumor specimens | 107 | NA | Clone SP76, BD Transduction Laboratories Ventana | Nuclear 1% | NA | Tumor grade, tumour size, pN, pT, ER, and HER2 status | DFS | 17 |
|  | Shen 2014 | 2014 | China | 45 months (2-85.5) | 51 | IHC, blind | Advanced cervical squamous cell carcinoma | Retrospective, multicentre | Paraffin-embedded tumor specimens | 132 | Radiotherapy | Santa Cruz Biotechnology Inc; Dallas, TX; dilution 1:100 | Nuclear ≥6 scores | 5 years | Age, FIGO stage, histopathological grade, tumor diameter, and combined chemotherapy | PFS | 25 |
|  | Udagawa 2015 | 2015 | Japan | 8.6 years (0.3-16.3) | 66 | IHC | Lung squamous cell carcinoma with lymph node metastasis | Retrospective, single-center | Paraffin-embedded tumor specimens | 113 | Surgery | Clone 3A2, Applied Biological Materials; dilution 1:150 | NA median | NA | Lymph node factor | RFS | 16 |
|  | Sodja 2016 | 2016 | Slovenia | 8.5 months (0.5-32.5) | 65 | qRT-PCR | Advanced small-cell lung cancer | Prospective, single-center | Blood | 50 | First-line chemotherapy | ABI PRISM 7500 FAST Real-Time PCR System | 0.8 | NA | Gender, age, PS, and the number of distant metastatic sites | DFS, PFS | 28 |
|  | Yamawaki 2017 | 2017 | Japan | NA | NA | IHC | Advanced endometrial cancer | Retrospective, single-center | Paraffin-embedded tumor specimens | 31 | NA | Cell Signaling Technology, Danvers, MA, USA; dilution 1:200 | Nuclear 10% | NA | Tumor grade, malignant ascites, ER, and PR | PFS | 14 |

NA: not applicable; NASBA: nuclear acid sequence-based amplification; IHC: immunohistochemistry; qRT-PCR: Real-Time Quantitative PCR; OS: overall survival; DFS: disease-free survival; PFS: progression-free survival; CSS: cancer-specific survival; RFS: recurrence-free survival (RFS); REMARK: Reporting Recommendations for Tumor Marker Prognostic Studies; LDH: lactate dehydrogenase; VEGF: vascular epithelial growth factor; EGFR: epidermal growth factor receptor; MVD: microvessel density; UICC: International Union Against Cancer; CNS: central nervous system; IVE: intravascular emboli; pN: lymph node; CEA: carcinoembryonic antigen; CA199: carbohydrate antigen 199; CA242: carbohydrate antigen 242; ER: estrogen receptor; HER2: human epidermalgrowth factor receptor-2; FIGO: International Federation of Gynecology and Obstetrics; PS: East Cooperative Oncology Group performance status.
