# Supplementary figures and images for: Prognostic Value of CD133 and SOX2 in Advanced Cancer
Source: J Oncol. 2019 Jan 1;2019:3905817. doi: 10.1155/2019/3905817 (PMC6332999; doi:10.1155/2019/3905817)

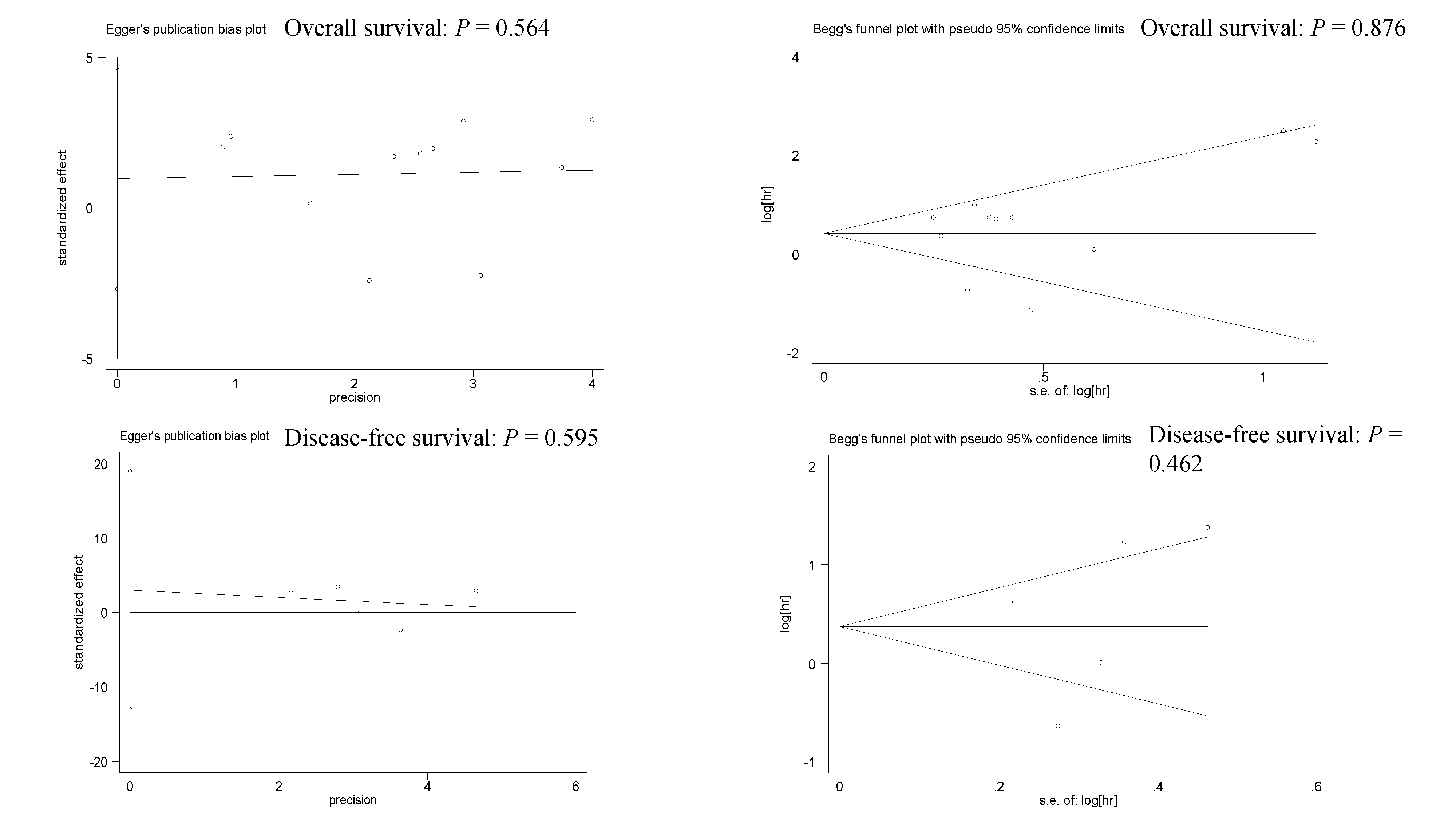

Supplement: Supplementary 3 — Figure S1: Publication bias using Egger's and Begg's tests for overall survival (OS) and disease-free survival (DFS) of CD133 positive expression. [file 3905817.f3.tiff]

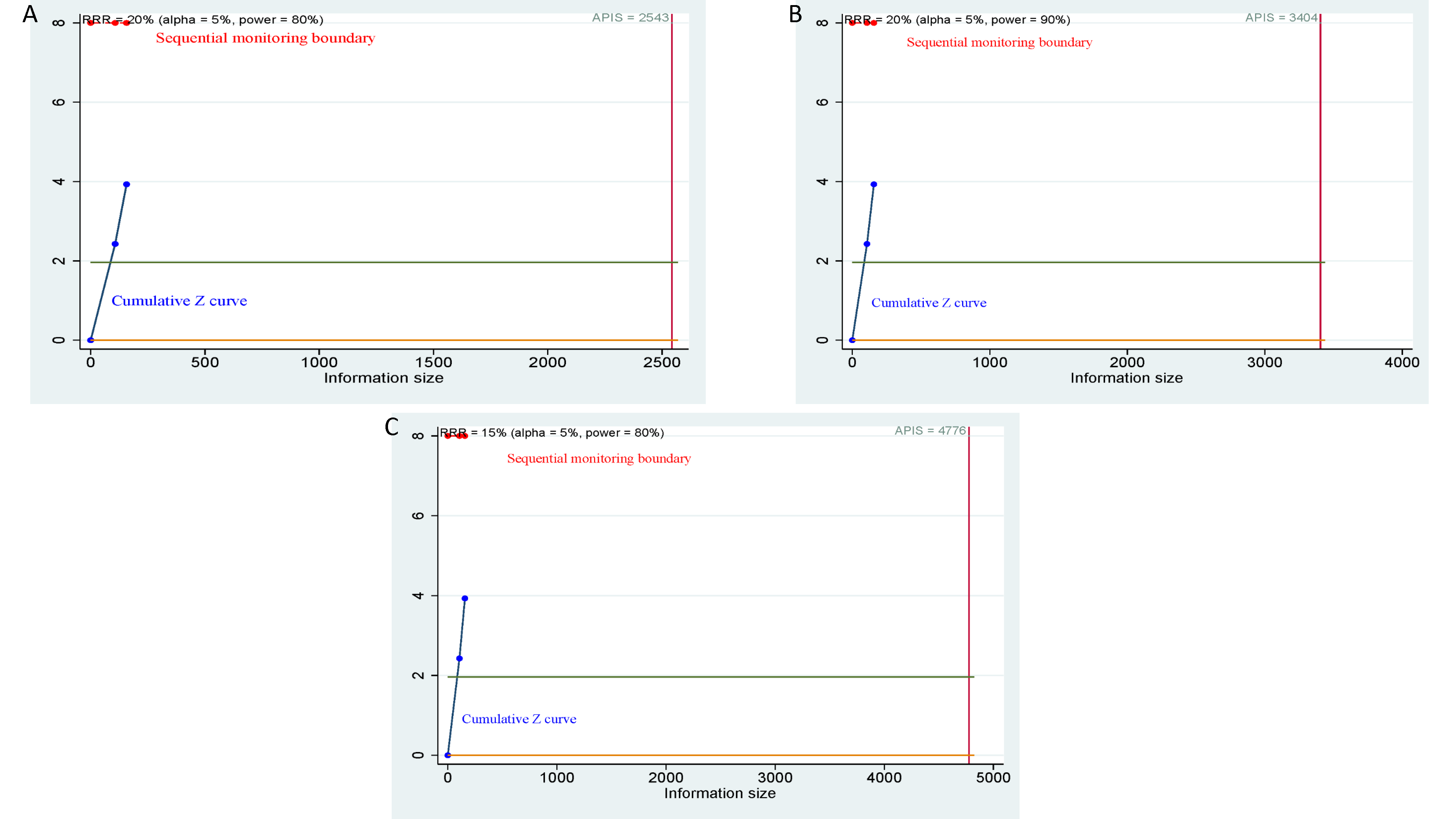

Supplement: Supplementary 4 — Figure S2: Trial sequential analysis (TSA) for disease-free survival (DFS) of SOX2 positivity. [file 3905817.f4.tif]
